# Prevalence of *Enterobius vermicularis* infections and associated risk factors among schoolchildren in Nakhon Si Thammarat, Thailand

**DOI:** 10.1186/s41182-020-00270-3

**Published:** 2020-09-29

**Authors:** Pokkamol Laoraksawong, Pimyada Pansuwan, Supakrit Krongchon, Pongphan Pongpanitanont, Penchom Janwan

**Affiliations:** 1 School of Health Science, Sukhothai Thammathirat Open University, Nonthaburi, Thailand; 2 Department of Medical Technology, School of Allied Health Sciences, Walailak University, Nakhon Si Thammarat, Thailand

**Keywords:** *Enterobius vermicularis*, Prevalence, Risk factors, Schoolchildren, Southern Thailand

## Abstract

**Background:**

*Enterobius vermicularis* infection is an important public health problem worldwide, especially among schoolchildren in tropical and subtropical countries. The prevalence of *E*. *vermicularis* infections varies in each region of Thailand; however, its status remains unknown among children who live in rural areas of the southern region. This study aimed to evaluate the current prevalence of *E*. *vermicularis* infections and to identify the risk factors for infection among schoolchildren who live in rural communities in Nakhon Si Thammarat, Southern Thailand.

**Results:**

The overall prevalence of *E*. *vermicularis* infections was 5.79% (23 of 397). According to a multivariate analysis, the following were found to be risk factors associated with *E*. *vermicularis* infections (*P* < 0.05): those of the male sex (AOR = 4.03, 95% CI 1.22–13.29), those in the 3–6 year age group (AOR = 4.85, 95% CI 1.51–15.59), those who have a mother with a primary school education level (AOR = 11.22, 95% CI 1.75–71.77), those who have older sibling(s) (AOR = 6.25, 95% CI 1.83–21.26), those who have younger sibling(s) (AOR = 6.24, 95% CI 2.00–19.44), those who sometimes wash their hands after using the toilet (AOR = 5.25, 95% CI 1.24–22.21), those who keep their fingernails long (AOR = 29.97, 95% CI 6.16–145.85), and those who suck their fingers (AOR = 3.59, 95% CI 1.21–10.66).

**Conclusions:**

This was the first report that revealed the prevalence of *E*. *vermicularis* infections among children who live in rural areas of Southern Thailand through detection using the Scotch tape technique. This study demonstrated that the high prevalence of *E*. *vermicularis* infections in schoolchildren with siblings was a significant independent predictor and that the transmission of this infection may occur in the family through their school-age siblings. In addition, children who have poor personal hygiene have a high prevalence of *E*. *vermicularis* infections. Therefore, maintaining good handwashing habits, keeping one’s fingernails short, and avoiding sucking one’s fingers should be important preventive measures against infection. Moreover, health literacy or health education, especially for parents or the principal caretakers of children, should be implemented to reduce *E*. *vermicularis* infections.

## Background

Enterobiasis is an intestinal nematode infection caused by *Enterobius vermicularis*, commonly known as pinworms. *E*. *vermicularis* infection is an important public health problem among schoolchildren, especially in tropical and subtropical countries [1, 2], with an estimate of over 1 billion infections [3]. Most of the infections are asymptomatic. Common enterobiasis symptoms include itching, irritation of the perianal region, and vaginal pruritus in females [4, 5]. In severe infection cases, the symptoms include insomnia, weight loss, vomiting, abdominal pain, and appendicitis [2, 6, 7]. *E*. *vermicularis* has a simple life cycle, where it is transmitted via the finger-oral route, inhalation, or reinfection [1, 6, 8].

In many parts of the world, the prevalence of *E*. *vermicularis* infections varies between 0.21 and 54.86% [1–3, 8–17]. In Thailand, the prevalence of *E*. *vermicularis* infections varies between 0 and 50.90% [18–30]. Schoolchildren who live in crowded environments and have poor personal hygiene are the most commonly infected group [10, 22]. Although various studies have been conducted on the distribution and prevalence of *E*. *vermicularis* infections in Thailand, epidemiological information on *E*. *vermicularis* infections is lacking for several remote regions, especially Southern Thailand. A national survey of helminthiasis in Thailand that was conducted in 2009 showed a prevalence rate of 0.2% for *E*. *vermicularis* infections in Southern Thailand [31]. A survey of intestinal parasitic infections in Nakhon Si Thammarat, Southern Thailand, that was conducted in 2016 showed a 0.3% prevalence rate for *E*. *vermicularis* infections [32]. However, these recent publications found *E*. *vermicularis* infections incidentally through methods of parasitological surveys that did not employ the Scotch tape technique, which is the gold standard for *E*. *vermicularis* detection. Consequently, this study aimed to evaluate the prevalence of *E*. *vermicularis* infections among schoolchildren in the Tha Sala District of Nakhon Si Thammarat using the Scotch tape technique and to identify the potential risk factors for infection in the study area. The results are important for monitoring and implementing effective control strategies, as well as for providing supplemental data to convince policymakers that this infection remains important.

## Methods

### Study design and area

**Fig. 1:**
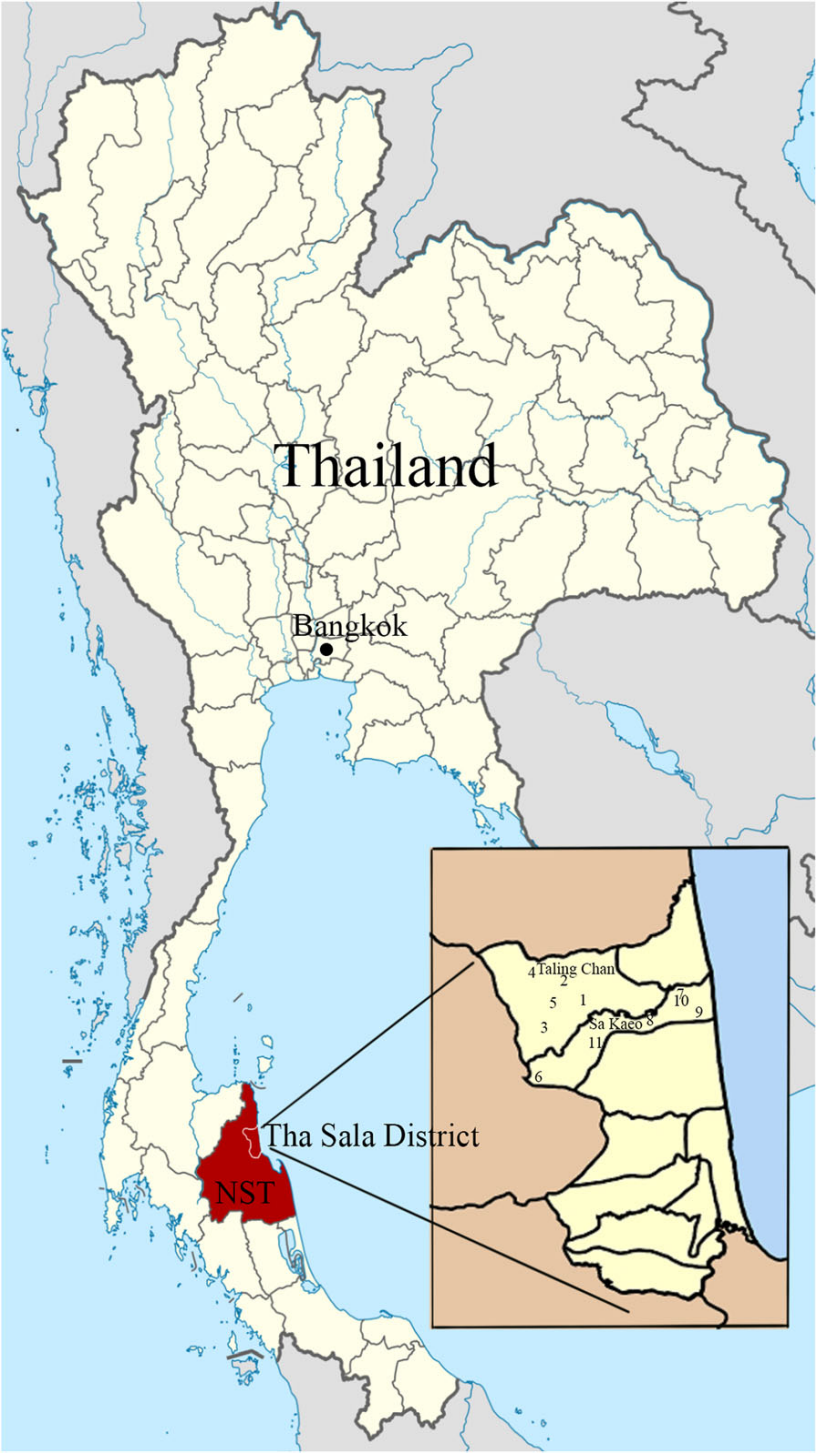
Map of the Taling Chan and Sa Kaeo subdistricts in the Tha Sala District, Nakhon Si Thammarat (NST) Province, Southern Thailand. Numbers 1 to 11 are the coordinates of each selected school in this study (map from Wikipedia: https://de.wikipedia.org/wiki/Datei:Thailand_Nakhon_Si_Thammarat_locator_map.svg and https://de.wikipedia.org/wiki/Amphoe_Tha_Sala)

A cross-sectional study was conducted from June to July 2019 and included schoolchildren living in rural areas of the Taling Chan and Sa Kaeo subdistricts of the Tha Sala District, Nakhon Si Thammarat Province, Southern Thailand. The study area is approximately located 754 km south of Bangkok, the capital city of Thailand. The average temperature is 27.3 °C, with a low of 23.7 °C and a high of 35.2 °C. The annual rainfall is 2150.0 mm [33]. The Taling Chan and Sa Kaeo subdistricts cover an area of 60.63 and 39.50 km^2^, respectively, with geographical locations at 8.770288 latitude and 99.885376 longitude, and 8.762168 latitude and 99.914407 longitude, respectively (Fig. 1). Both the Taling Chan and Sa Kaeo subdistricts are similar in terms of topography, climate, natural resources, land use, culture, and economic status.

### Study population, sample size, and sampling technique

Schoolchildren from 3 to 9 years of age who agreed to participate and whose parents or legal guardians gave consent were included. The sample size was determined using the finite single population proportion formula as follows [34]:

$$ n=\frac{Np\left(1-p\right){Z}_{1-\alpha /2}^2}{d^2\left(N-1\right)+p\left(1-p\right){Z}_{1-\alpha /2}^2} $$

The sample size was calculated using a prevalence rate (*p*) of 39.0%, as detailed in a previous study [20], with a 95% confidence interval (95% CI) (*z* = 1.96) and a 5% margin of error (*d* = 0.05). The population of the students from 3 to 9 years of age in Tha Sala District totaled 5412 persons [35]. The calculated sample size was 343 people. The final sample size was 378 individuals, including a 10% nonresponse rate among the students. The participants were randomly selected from all 11 kindergarten and primary schools within the Taling Chan and Sa Kaeo subdistricts using a voluntary sampling method.

### Parasitological survey and data collection

Letters of information, informed consent forms, and self-administered questionnaires were given to parents or legal guardians prior to pinworm screening. Parents and children were informed about the timing of the experiment in advance. The collection of samples was performed in the morning before the defecating and bathing of the children. Children were diagnosed for *E*. *vermicularis* infections based on the Scotch tape technique [6]. Only one sample was taken from each child, and the microscopic examinations were performed by two medical technologists. The child’s parents or principal caretakers were asked to complete a questionnaire that inquired about the potential risk factors involved. The questionnaire (Additional file 1) was developed and used to access data such as information related to demographics, personal hygiene, and household sanitary conditions. We collected information on the child’s gender, the child’s age, the number of household members, the presence of older/younger sibling(s), the parents’ education level, the parents’ occupation, the parents’ income, handwashing behavior, fingernail trimming, finger sucking, playing with others, bathing behavior, underwear washing, towel or bed-sharing, anthelmintic medication, living conditions, and household cleaning.

### Data analysis

The total data were cleaned, entered, and analyzed using STATA version 10.1 (STATA Corp., TX, USA). The demographic characteristics of the participants were described using frequencies, percentages, and 95% CI for categorical data; means and standard deviations (SD) were used for continuous data. To investigate factors that affected *E*. *vermicularis* infections, the prevalence rate, 95% CI, and odds ratios (ORs) were estimated using simple and multiple logistic regressions and a generalized estimating equation (GEE). To adjust for possible confounders, all variables with a *P* value less than 0.2 in the univariate analysis were selected. A *P* value of less than 0.05 was considered statistically significant.

## Results

### Demographic characteristics

From a total of 859 schoolchildren (aged 3–9 years in 2 subdistricts) included in the study, 397 and their parents responded, making the response rate 46.2%. In total, 397 children were enrolled in this study; 205 (51.64%) of the children were girls and 192 (48.36%) were boys. The mean (± SD) age of the children was 6.79 (± 1.74) years (Additional file 2: Table S1).

### Personal hygiene behaviors

Two hundred and forty-nine (62.72%) of the children reported sometimes washing their hands after using toilet facilities. Three hundred and eighty-one (95.97%) of children reported keeping their fingernails short, and 332 (83.63%) of the children reported not sucking their fingers (Additional file 3: Table S2). Univariate analyses of personal hygienic factors indicated that there were no significant associations between pinworm infection and washing ones’ hands before eating, taking a bath before sleeping or after waking up, bathing without the help of family members, or washing one’s underwear without the help of family members (*P* > 0.05) (Additional file 3: Table S2). However, a significant higher risk of pinworm infection was found in the children who reported sometimes washing their hands after using toilet facilities, keeping their fingernails long, sucking their fingers, and sharing towels with others (*P* < 0.05) (Additional file 3: Table S2).

### Household sanitary conditions

Three hundred and eighty-eight (97.73%) children reported living in a single-family detached home. One hundred and fifty-nine (40.05%) families reported changing their bedding once every 2 weeks, and three hundred and twenty-five (81.86%) reported always cleaning their house (Additional file 4: Table S3). Univariate analyses of household sanitary conditions indicated that there were no significant associations between pinworm infection and the style of residence, the home structure, the type of bed, and the frequency of changing the bedding or cleaning the house (*P* > 0.05) (Additional file 4: Table S3).

### Prevalence of *E*. *vermicularis* infections

The overall prevalence of *E*. *vermicularis* infections through detection using the Scotch tape technique was 5.79% (95% CI 3.48–8.10). This prevalence rate of infection was higher in boys (7.29%) than in girls (4.39%). The egg-positive rate ranged from 0 to 15.79% (95% CI 0.99–1.01, *P* = 0.72) by location. No significant differences in egg-positive rates according to school were observed (Table 1).

**Table 1:** Prevalence of *Enterobius vermicularis* infection among the study participants from the 11 kindergarten and primary schools

School	Number examination	Number positive	Positive (%)
1	58	2	3.45
2	32	0	0
3	19	3	15.79
4	29	1	3.45
5	46	5	10.87
6	37	4	10.81
7	42	3	7.14
8	41	1	2.44
9	19	0	0
10	43	1	2.32
11	31	3	9.68

### Factors associated with *E*. *vermicularis* infections

According to the multivariate analysis, gender, age group, mother’s education level, having older sibling(s), having younger sibling(s), washing one’s hands after using toilet facilities, keeping one’s fingernails short, and sucking one’s fingers were found to be risk factors associated with *E*. *vermicularis* infections (*P* < 0.05). Boys were 4.03 times more likely (AOR = 4.03, 95% CI 1.22–13.29) to be infected than girls. Meanwhile, children 3 to 6 years of age were 4.85 times more likely (AOR = 4.85, 95% CI 1.51–15.59) to be infected than children 7 to 9 years of age. Children who had a mother with primary school education level were 11.22 times more at risk (AOR = 11.22, 95% CI 1.75–71.77) to be infected than those who had a mother with a higher level of education. Moreover, children who had older sibling(s) were 6.25 times more likely (AOR = 6.25, 95% CI 1.83–21.26) to be infected than children who did not have older siblings. Additionally, children who had younger sibling(s) were 6.24 times (AOR = 6.24, 95% CI 2.00–19.44) more likely to be infected than children who did not have younger sibling(s). Furthermore, children who did not frequently wash their hands after using toilet facilities were 5.25 times more likely (AOR = 5.25, 95% CI 1.24–22.21) to be infected than those who did wash their hands. Children who kept their fingernails long were 29.97 times more likely (AOR = 29.97, 95% CI 6.16–145.85) to be infected than those who kept them short. Additionally, children who sucked their fingers were 3.59 times more likely (AOR = 3.59, 95% CI 1.21–10.66) to be infected than those who did not suck them (Table 2).

**Table 2:** Multivariate analysis of risk factors associated with *Enterobius vermicularis* infections among the study participants

Characteristics	Total number	Number positive (PR^**a**^)	COR^**b**^	AOR^**c**^ (95% CI^**d**^)	***P*** value
**Gender**					
Female	205	9 (4.4)	1	1	0.022*
Male	192	14 (7.3)	1.7	4.03 (1.2–13.3)	
**Age group**					
7 to 9 years	242	10 (4.1)	1	1	0.008*
3 to 6 years	155	13 (8.4)	2.1	4.85 (1.5–15.6)	
**Mother’s education level**					
Diploma, bachelor’s, or higher	56	2 (3.6)	1	1	0.016*
Secondary school	215	13 (6.1)	1.7	4.11 (0.7–22.6)	
Primary school	126	8 (6.4)	1.8	11.22 (1.8–71.8)	
**Have older sibling(s)**					
No	184	5 (2.7)	1	1	0.003*
Yes	213	18 (8.5)	3.3	6.25 (1.8–21.3)	
**Have younger sibling(s)**					
No	270	11 (4.1)	1	1	0.002*
Yes	127	12 (9.5)	2.5	6.24 (2.0–19.4)	
**Wash hands after using toilet facilities**					
Always	148	3 (2.0)	1	1	0.024*
Sometimes	249	20 (8.0)	4.2	5.25 (1.2–22.2)	
**Keep fingernails short**					
Yes	381	16 (4.2)	1	1	< 0.001*
No	16	7 (43.8)	17.7	29.97 (6.2–145.9)	
**Sucks fingers**					
No	332	12 (3.6)	1	1	0.022*
Yes	65	11 (16.9)	5.4	3.59 (1.2–10.7)	

*Significant association

^a^*PR* prevalence rate in each group

^b^*COR* crude odds ratio by univariable analysis

^c^*AOR* adjusted odds ratio by multivariable analysis

^d^*CI* 95% confidence interval

## Discussion

This is the first report that reveals the prevalence of *E*. *vermicularis* infections among children who live in rural areas of Southern Thailand through detection using the Scotch tape technique. In this study, the overall prevalence of *E*. *vermicularis* infections was 5.79%, lower than the values found in previous studies that have been conducted in other regions of Thailand. This discrepancy may partly be due to the study setting and the source population difference. The prevalence of pinworm infections ranges from 7.81 to 38.82% [19–21, 27, 28, 30], 11.30 to 50.90% [18, 29], and 7.25 to 45.38% [22–25] in the central, northeast, and northern regions of Thailand, respectively. However, according to these studies, the prevalence of *E*. *vermicularis* infections in Thailand tends to be lower over the past three decades mainly due to sanitary environments and healthcare access being greatly improved by urbanization.

Our study revealed that boys are more highly infected with *E*. *vermicularis* than girls, which was also observed in previous studies [9, 12]. Higher infection rates among boys may be due to boys being involved in more activities, being in closer contact with other children, and having poorer personal hygiene than girls. Among the age groups, children 3 to 6 years of age are more at risk to be infected with *E*. *vermicularis*, which is consistent with the findings of previous studies [13, 29, 30]. This could be because this age group engages in more group activities together, such as taking naps together during the day on floor mats, and because they have poorer personal hygiene than older children.

The three main factors that are often involved in *E*. *vermicularis* infections among children are family background, living conditions, and personal hygiene. In the present study, children with mothers who have a primary school level of education have a higher risk of infection. This finding is similar to those reported previously [1, 13], and it may be explained by the fact that mothers with low education levels may not have accurate knowledge of pinworm infection even though they are the principal caretaker of their children. Meanwhile, previous reports have suggested that parents’ knowledge about enterobiasis might be one of the most important risk factors for enterobiasis in children [11, 14]. In addition, children who have younger/older siblings are more highly infected with *E*. *vermicularis* than those who do not, and these findings are similar to those reported previously [1, 12, 17]. These results suggest that new infection or reinfection may occur in the family among children who are in constant close contact over long periods of time. Further, it seems that residents, including parents or caretakers, should also be investigated and treated. The medications used for the treatment of pinworm are either albendazole, mebendazole, or pyrantel pamoate. Single doses of albendazole (400 mg), mebendazole (100 mg), or pyrantel pamoate (11 mg/kg up to 1 g) are highly effective. A second dose is given 2 weeks later because of the frequency of reinfection and autoinfection. Repeated infections should be treated using the same method as that used during the first infection. In households where more than one member is infected or where infections are repeated, symptomatic infections occur; thus, it is recommended that all household members be treated at the same time. In institutions, a mass simultaneous treatment that is repeated in 2 weeks can be effective [4, 6, 36]. The eggs are very lightweight and can thus be distributed into the surrounding environment. Dust-borne infection; indirect infection through clothing, bedding, or food; or direct infection by hand from the anus to the mouth is possible, particularly for children in rural areas with inadequate personal hygiene, especially those who engage in fingernail sucking or biting and do not frequently wash their hands. Prophylaxis is dependent primarily on personal hygiene behaviors and household sanitary conditions [4, 6].

Previous reports have suggested that inadequate personal hygiene can increase the risk of enterobiasis in children [1, 11, 13–15]. Our study shows that children who do not frequently wash their hands after using toilet facilities are more at risk to be infected with pinworms. This finding is similar to those reported previously [1, 14], indicating that direct infection by hand from the anus to the mouth might occur. Additionally, it also causes distribution into the surrounding environment. Moreover, children who suck their fingers are more at risk to be infected with *E*. *vermicularis*, which is consistent with the findings of other studies [11, 15]. Interestingly, we found that children who keep their fingernails long are more at risk to be infected with pinworms, which is consistent with the findings of a previous study [11]. This could be due to pinworm eggs that may be transferred from contaminated hands to one’s mouth. Finally, in our study, these findings indicate that the finger-oral route remains the most important avenue for *E*. *vermicularis* transmission.

One limitation in our study was related to using a single-day Scotch tape technique for parasite examination, which is not valid enough to measure the true prevalence of pinworm infection. The prevalence demonstrated in this study was underestimated. Another important limitation was that the causes and risk factors associated with *E*. *vermicularis* infection might not be strongly demonstrated from the cross-sectional design of the study.

## Conclusion

This current study demonstrated that having siblings as a schoolchild is a significant independent predictor of the high prevalence of *E*. *vermicularis* infections and that the transmission of this infection may occur in the family through school-age siblings. In addition, children who have poor personal hygiene have a high prevalence of *E*. *vermicularis* infections. Therefore, good handwashing habits, keeping one’s fingernails short, and avoiding sucking fingers should be important preventive measures against the infection. Moreover, health literacy or health education, especially for parents or the principal caretakers of children, should be implemented to reduce *E*. *vermicularis* infections. Additionally, these results should encourage policymakers and public health personnel to improve programs for pinworm control and health promotion.

## Supplementary information

**Additional file 1.** Questionnaire for demographic, personal hygiene, and household sanitary conditions data collection.

**Additional file 2: Table S1.** Univariate analysis of the demographic characteristics of the study participants.

**Additional file 3: Table S2.** Univariate analysis of personal hygiene factors associated with *Enterobius vermicularis* infections among the study participants.

**Additional file 4: Table S3.** Univariate analysis of household sanitary conditions associated with *Enterobius vermicularis* infections among the study participants.

## Data Availability

All data generated or analyzed during this study are included in this published article and its supplementary information files. The raw data are available from the corresponding author on reasonable request.

## Abbreviations

CI: Confidence interval
SD: Standard deviation
THB: Thai baht
OR: Odds ratio
COR: Crude odds ratio
AOR: Adjusted odds ratio
