# Supplementary material for: Prevalence of Enterobius vermicularis infections and associated risk factors among schoolchildren in Nakhon Si Thammarat, Thailand
Source: Trop Med Health. 2020 Sep 29;48:83. doi: 10.1186/s41182-020-00270-3 (PMC7523320; doi:10.1186/s41182-020-00270-3)
Supplement: Supplementary file 1 — Additional file 1. Questionnaire for demographic, personal hygiene, and household sanitary conditions data collection. [file 41182_2020_270_MOESM1_ESM.docx]

**Code ............................**

**Questionnaire**

**Part 1: General information**

1. Gender of child: □ Female □ Male

2. Age of child: ............. years

3. Number of family members in your household: ............. persons

4. Does your child have older brother(s) or sister(s): □ No □ Yes

5. Does your child have younger brother(s) or sister(s): □ No □ Yes

6. Father’s education level: □ Diploma, bachelor or higher

□ Secondary school □ Primary school

7. Mother’s education level: □ Diploma, bachelor or higher

□ Secondary school □ Primary school

8. Father’s occupation:

□ Agriculture □ Trade/business owner □ Government/private officer

9. Mother’s occupation:

□ Agriculture □ Trade/business owner □ Government/private officer

10. Father’s income: □ < 2,000 ฿ □ 2,000–4,999 ฿

□ 5,000–9,999 ฿ □ ≥ 10,000 ฿

11. Mother’s income: □ < 2,000 ฿ □ 2,000–4,999 ฿

□ 5,000–9,999 ฿ □ ≥ 10,000 ฿

**Part 2: Personal hygiene and possible risk factors**

1. Does your child wash their hands before eating?

□ Always □ Sometimes

2. Does your child wash their hands after using toilet facilities?

□ Always □ Sometimes

3. Does your child keep their fingernails short? □ Yes □ No

4. Does your child suck their fingers? □ Yes □ No

5. Does your child play with others? □ Yes □ No

6. Does your child take a bath before sleeping? □ Yes □ No

7. Does your child take a bath after waking up? □ Yes □ No

8. Does your child bathe without the help of family members? □ Yes □ No

9. Does your child wash their underwear without the help of family members?

□ Yes □ No

10. Does your child share a towel with others? □ Yes □ No

11. Does your child share a bed with family members? □ Yes □ No

12. Has your child ever been dewormed of parasites? □ Yes □ No

Part 3: Household sanitary conditions and possible risk factors

1. What is the style of your residence?

□ Single-family detached home □ Apartment

2. What is the structure of your home made of? □ Concrete □ Wood

3. What type of bed does your child have?

□ Wood or spring mattress □ Floor mat

4. How often do you change your bedding?

□ Once a week □ Once every two weeks □ Once a month or more

5. Do you clean your house every day? □ Yes □ No

**Thank you for your kind cooperation.**
