# Supplementary material for: Prevalence of Enterobius vermicularis infections and associated risk factors among schoolchildren in Nakhon Si Thammarat, Thailand
Source: Trop Med Health. 2020 Sep 29;48:83. doi: 10.1186/s41182-020-00270-3 (PMC7523320; doi:10.1186/s41182-020-00270-3)
Supplement: Supplementary file 2 — Additional file 2: Table S1. Univariate analysis of the demographic characteristics of the study participants. [file 41182_2020_270_MOESM2_ESM.docx]

**Additional file 2: Table S1.** Univariate analysis of the demographic characteristics of the study participants

| **Characteristic** | **Number (%)** | **Number positive (PR^a^)** | **COR^b^ (95% CI^c^)** | **P-value** |
| --- | --- | --- | --- | --- |
| **Gender of children** |  |  |  |  |
| Female | 205 (51.6) | 9 (4.4) | 1 | 0.210 |
| Male | 192 (48.4) | 14 (7.3) | 1.71 (0.7-4.1) |  |
| **Age group** |  |  |  |  |
| 7 to 9 years | 242 (61.0) | 10 (4.1) | 1 | 0.081 |
| 3 to 6 years | 155 (39.0) | 13 (8.4) | 2.12 (0.9-5.0) |  |
| Mean ± SD | 6.8 ± 1.7 |  |  |  |
| Min:Max | 3:9 |  |  |  |
| **Number of family members** |  |  |  |  |
| < 5 | 185 (46.6) | 9 (4.9) | 1 | 0.450 |
| ≥ 5 | 212 (53.4) | 14 (6.6) | 1.38 (0.6-3.3) |  |
| **Have older sibling(s)** |  |  |  |  |
| No | 184 (46.3) | 5 (2.7) | 1 | 0.010* |
| Yes | 213 (53.7) | 18 (8.5) | 3.3 (1.2-9.1) |  |
| **Have younger sibling(s)** |  |  |  |  |
| No | 270 (68.0) | 11 (4.1) | 1 | 0.030* |
| Yes | 127 (32.0) | 12 (9.5) | 2.45 (1.1-5.7) |  |
| **Father’s education level** |  |  |  |  |
| Diploma, bachelor’s or higher | 86 (21.7) | 2 (2.3) | 1 | 0.120 |
| Secondary school | 226 (56.9) | 13 (5.8) | 2.56 (0.6-11.6) |  |
| Primary school | 85 (21.4) | 8 (9.4) | 4.36 (0.9-21.2) |  |
| **Mother’s education level** |  |  |  |  |
| Diploma, bachelor’s or higher | 56 (14.1) | 2 (3.6) | 1 | 0.710 |
| Secondary school | 215 (54.2) | 13 (6.0) | 1.73 (0.4-7.9) |  |
| Primary school | 126 (31.7) | 8 (6.4) | 1.83 (0.4-8.9) |  |
| **Father’s occupation** |  |  |  |  |
| Agriculture | 151 (38.0) | 7 (4.6) | 1 | 0.730 |
| Trade/business owner | 214 (53.9) | 14 (6.5) | 1.44 (0.6-3.7) |  |
| Government/private officer | 32 (8.1) | 2 (6.3) | 1.13(0.3-6.9) |  |
| **Mother’s occupation** |  |  |  |  |
| Agriculture | 111 (28.0) | 6 (5.4) | 1 | 0.560 |
| Trade/business owner | 243 (61.2) | 17 (7.0) | 1.31 (0.5-3.4) |  |
| Government/private officer | 43 (10.8) | 0 (0.0) | Omitted |  |
| **Father’s income** |  |  |  |  |
| ≥ 10000 THB^d^ | 115 (29.0) | 6 (5.2) | 1 | 0.750 |
| < 10000 THB | 282 (71.0) | 17 (6.0) | 1.16 (0.4-3.0) |  |
| **Mother’s** **income** |  |  |  |  |
| ≥ 10000 THB | 66 (16.6) | 1 (1.5) | 1 | 0.060 |
| < 10000 THB | 331 (83.4) | 22 (6.7) | 4.63 (0.6-34.9) | |

* Significant association

^a^ PR: Prevalence rate in each group

^b^ COR: Crude odds ratio by univariable analysis

^c^ CI: 95% Confidence interval

^d^ THB: Thai baht
