# Supplementary material for: Prevalence of Enterobius vermicularis infections and associated risk factors among schoolchildren in Nakhon Si Thammarat, Thailand
Source: Trop Med Health. 2020 Sep 29;48:83. doi: 10.1186/s41182-020-00270-3 (PMC7523320; doi:10.1186/s41182-020-00270-3)
Supplement: Supplementary file 3 — Additional file 3: Table S2. Univariate analysis of personal hygiene factors associated with Enterobius vermicularis infections among the study participants. [file 41182_2020_270_MOESM3_ESM.docx]

**Additional file 3: Table S2.** Univariate analysis of personal hygiene factors associated with *Enterobius vermicularis* infections among the study participants

| **Characteristics** | **Number (%)** | **Number positive (PR^a^)** | **COR^b^ (95% CI^c^)** | **P-value** |
| --- | --- | --- | --- | --- |
| **Wash hands before eating** |  |  |  |  |
| Always | 74 (18.6) | 3 (4.1) | 1 | 0.459 |
| Sometimes | 323 (81.4) | 20 (6.2) | 1.56 (0.5-5.4) |  |
| **Wash hands after using toilet facilities** |  |  |  |  |
| Always | 148 (37.3) | 3 (2.0) | 1 | 0.007* |
| Sometimes | 249 (62.7) | 20 (8.0) | 4.22 (1.2-14.5) | |
| **Keep fingernails short** |  |  |  |  |
| Yes | 381 (96.0) | 16 (4.2) | 1 | <0.001* |
| No | 16 (4.0) | 7 (43.8) | 17.74 (5.9-53.7) | |
| **Sucks fingers** |  |  |  |  |
| No | 332 (83.6) | 12 (3.6) | 1 | <0.001* |
| Yes | 65 (16.4) | 11 (16.9) | 5.43 (2.3-12.9) | |
| **Play with others** |  |  |  |  |
| No | 8 (2.0) | 0 (0.0) | Omitted |  |
| Yes | 389 (98.0) | 23 (5.9) |  |  |
| **Take a bath before sleeping** |  |  |  |  |
| Yes | 382 (96.2) | 21 (5.5) | 1 | 0.270 |
| No | 15 (3.8) | 2 (13.3) | 2.64 (0.6-12.5) | |
| **Take a bath after waking up** |  |  |  |  |
| Yes | 383 (96.5) | 21 (5.5) | 1 | 0.230 |
| No | 14 (3.5) | 2 (14.3) | 2.87 (0.6-13.7) | |
| **Bath without the help of family members** | |  |  |  |
| No | 122 (30.7) | 4 (3.3) | 1 | 0.130 |
| Yes | 275 (69.3) | 19 (6.9) | 2.19 (0.7-6.6) |  |
| **Wash underwear without the help of family members** |  |  |  |  |
| No | 353 (88.9) | 20 (5.7) | 1 | 0.760 |
| Yes | 44 (11.1) | 3 (6.8) | 1.22 (0.3-4.3) |  |
| **Share a towel with others** |  |  |  |  |
| No | 316 (79.6) | 13 (4.1) | 1 | 0.009* |
| Yes | 81 (20.4) | 10 (12.4) | 3.28 (1.4-7.8) |  |
| **Share a bed with family members** |  |  |  |  |
| No | 15 (3.8) | 0 (0.0) | Omitted |  |
| Yes | 382 (96.2) | 23 (6.0) |  |  |
| **Deworm parasite** |  |  |  |  |
| Yes | 59 (14.9) | 0 (0.0) | Omitted |  |
| No | 338 (85.1) | 23 (6.0) |  |  |

* Significant association

^a^ PR: Prevalence rate in each group

^b^ COR: Crude odds ratio by univariable analysis

^c^ CI: 95% Confidence interval
