# Supplementary material for: Prevalence of Enterobius vermicularis infections and associated risk factors among schoolchildren in Nakhon Si Thammarat, Thailand
Source: Trop Med Health. 2020 Sep 29;48:83. doi: 10.1186/s41182-020-00270-3 (PMC7523320; doi:10.1186/s41182-020-00270-3)
Supplement: Supplementary file 4 — Additional file 4: Table S3. Univariate analysis of household sanitary conditions associated with Enterobius vermicularis infections among the study participants. [file 41182_2020_270_MOESM4_ESM.docx]

**Additional file 4: Table S3.** Univariate analysis of household sanitary conditions associated with *Enterobius vermicularis* infections among the study participants

| **Characteristics** | **Number (%)** | **Number positive (PR^a^)** | **COR^b^ (95% CI^c^)** | **P-value** | | |
| --- | --- | --- | --- | --- | --- | --- |
| **Style of residence** |  |  |  | |  |  |
| Single-family detached | 388 (97.7) | 22 (5.7) | 1 | | 0.530 |  |
| Apartment | 9 (2.3) | 1 (11.1) | 2.07 (0.2-17.4) | |  |  |
| **Home's structure** |  |  |  | |  |  |
| Concrete | 317 (79.9) | 15 (4.7) | 1 | | 0.090 |  |
| Wood | 80 (20.1) | 8 (10.0) | 2.24 (0.9-5.5) | |  |  |
| **Type of bed** |  |  |  | |  |  |
| Wood or spring mattress | 153 (38.5) | 7 (4.6) | 1 | | 0.400 |  |
| Floor mat | 244 (61.5) | 16 (6.6) | 1.46 (0.6-3.6) | |  |  |
| **Frequency of changing bedding** |  |  |  | |  |  |
| Once a week | 148 (37.3) | 8 (5.4) | 1 | | 0.360 |  |
| Once every two weeks | 159 (40.0) | 12 (7.6) | 1.42 (0.6-3.6) | |  |  |
| Once a month or more | 90 (22.7) | 3 (3.3) | 0.6 (0.2-2.3) | |  |  |
| **Cleaning house every day** |  |  |  | |  |  |
| Yes | 325 (81.9) | 16 (4.9) | 1 | | 0.130 |  |
| No | 72 (18.1) | 7 (9.7) | 2.08 (0.8-5.3) | |  |  |

^a^ PR: Prevalence rate in each group

^b^ COR: Crude odds ratio by univariable analysis

^c^ CI: 95% Confidence interval
